# Structural network disruption markers explain disability in multiple sclerosis

**DOI:** 10.1136/jnnp-2018-318440

**Published:** 2018-11-22

**Authors:** Thalis Charalambous, Carmen Tur, Ferran Prados, Baris Kanber, Declan T Chard, Sebastian Ourselin, Jonathan D Clayden, Claudia A M Gandini Wheeler-Kingshott, Alan J Thompson, Ahmed T Toosy

**Affiliations:** 1 Department of Neuroinflammation, UCL Institute of Neurology, Queen Square MS Centre, London, UK; 2 Translational Imaging Group, Centre for Medical Image Computing (CMIC), Department of Medical Physics and Bioengineering, University College London, London, UK; 3 UCL GOS Institute of Child Health, University College London, London, UK; 4 Brain MRI 3T Research Center, C. Mondino National Neurological Institute, Pavia, Italy; 5 Department of Brain and Behavioural Sciences, University of Pavia, Pavia, Italy

**Keywords:** multiple sclerosis, mri, network analysis, EDSS, SDMT

## Abstract

**Objective:**

To evaluate whether structural brain network metrics correlate better with clinical impairment and information processing speed in multiple sclerosis (MS) beyond atrophy measures and white matter lesions.

**Methods:**

This cross-sectional study included 51 healthy controls and 122 patients comprising 58 relapsing–remitting, 28 primary progressive and 36 secondary progressive. Structural brain networks were reconstructed from diffusion-weighted MRIs and standard metrics reflecting network density, efficiency and clustering coefficient were derived and compared between subjects’ groups. Stepwise linear regression analyses were used to investigate the contribution of network measures that explain clinical disability (Expanded Disability Status Scale (EDSS)) and information processing speed (Symbol Digit Modalities Test (SDMT)) compared with conventional MRI metrics alone and to determine the best statistical model that explains better EDSS and SDMT.

**Results:**

Compared with controls, network efficiency and clustering coefficient were reduced in MS while these measures were also reduced in secondary progressive relative to relapsing–remitting patients. Structural network metrics increase the variance explained by the statistical models for clinical and information processing dysfunction. The best model for EDSS showed that reduced network density and global efficiency and increased age were associated with increased clinical disability. The best model for SDMT showed that lower deep grey matter volume, reduced efficiency and male gender were associated with worse information processing speed.

**Conclusions:**

Structural topological changes exist between subjects’ groups. Network density and global efficiency explained disability above non-network measures, highlighting that network metrics can provide clinically relevant information about MS pathology.

## Introduction

Multiple sclerosis (MS) is a chronic disease of the central nervous system. Inflammation and demyelination are predominant in relapsing–remitting MS (RRMS), while neurodegeneration is more prominent in the progressive phases (primary progressive MS (PPMS), secondary progressive MS (SPMS)).^1^ Because measures obtained through conventional MRI techniques show incomplete correlation with patients’ disability,^2^ more advanced techniques have been used demonstrating that grey matter (GM) atrophy^3^ and abnormalities outside white matter (WM) lesions^4^ also relate to cognitive dysfunction. A very common cognitive domain affected is information processing speed and is assessed by Symbol Digit Modalities Test (SDMT).^5^ Neurological impairment with particular emphasis on ambulation status is evaluated by another widely used measure, the Expanded Disability Status Scale (EDSS).^6^

Brain network analysis has been used to study topological alterations in pathology.^7^ For MS, diffusion-derived networks have shown reduced efficiency correlating with physical disability^8^ and network changes that suggest adaptations to preserve cognitive function.^9^ Whether network metrics explain disability beyond routine imaging metrics is unknown. Only one study addressed this but using only motor network efficiency.^10^ Additionally, network reconstruction techniques have not addressed tractogram biases.^11^ Recent technical work has improved the biological accuracy of streamline tractography,^12–14^ highlighting the necessity of state-of-the-art techniques in network studies. To our knowledge, these techniques have not yet been applied to MS.

In this cross-sectional study, using advanced network reconstruction methods, we aimed (1) to compare structural networks between study subgroups, (2) to investigate whether network metrics explain EDSS and SDMT above conventional MRI metrics, and (3) to determine the best statistical model that explains better EDSS and SDMT.

## Methods

### Participants

We recruited 122 patients with MS (58 RRMS, 28 PPMS and 36 SPMS) who had not experienced relapses within the preceding 4 weeks and classified as per Lublin and Reingold criteria.^15^ Fifty-one healthy controls (HC) were also examined. Participants underwent MRI and neurological assessment using EDSS.^6^ Verbal SDMT was performed in a subset of MS participants (n=60) (online supplementary etable 1) to screen for information processing speed. Fatigue (visual analogue scale), depression and anxiety (Hospital Anxiety and Depression Scale (HADS)) were also assessed in some patients (online supplementary etable 2).

10.1136/jnnp-2018-318440.supp2 Supplementary data

### MRI data acquisition

MRI data were acquired on a Philips Achieva 3T MR scanner (Philips Healthcare, Best, Netherlands) with a 32-channel head coil using (1) 3D sagittal T1-weighted scans with a fast-field echo scan, (2) whole brain High Angular Resolution Diffusion Imaging scan with echo planar imaging consisted of a cardiac-gated spin-echo sequence and (3) dual-echo proton density/T2-weighted axial oblique scans. All data were acquired with slices aligned with the anterior commisure - posterior commisure (AC-PC) line to minimise the effect of head positioning on data analysis.

### Structural imaging processing

A non-rigid transformation was performed to register the subject’s non-filled T1-weighted bias-field corrected image to the corresponding diffusion-weighted image (DWI) using BrainSuite V.15b^16^ resulting in a structural image of resolution 2×2×2 mm^3^. The lesion-filled T1-weighted images^17^ were then segmented into different tissue types and parcellated according to Desikan-Killiany-Tourville atlas protocol using GIF.^18^ The volumes of the various tissue types were estimated (normal-appearing brain volume (NABV), GM, cortical GM (CGM), deep GM (DGM)). Reduction of these volumes reflects atrophy. Lesion load (LL) was also computed as a measure of WM damage.

### Diffusion-weighted imaging processing and tractography

The mean b0 image was rigid registered to the first b0 image. Then, the same rigid transformation was applied to the 61 DWI volumes. FSL V.5.0.9 was used on the DWI data to correct for eddy currents and head motion^19^ and BrainSuite V.15b to correct for Echo-planar imaging (EPI) distortions using the T1-weighted image as the registration template for the diffusion data.^16^ For probabilistic tractography, we used second-order integration over fibre orientation distributions (iFOD2) estimated with constrained spherical deconvolution (CSD).^20^ A total of 10^7^ streamlines were generated implementing the anatomically constrained tractography (ACT) algorithm^12^ followed by spherical-deconvolution informed filtering of tractograms (SIFT2)^13^ (MRtrix3 V.0.3.14 package).

## Network reconstruction and metrics

**Figure 1:**
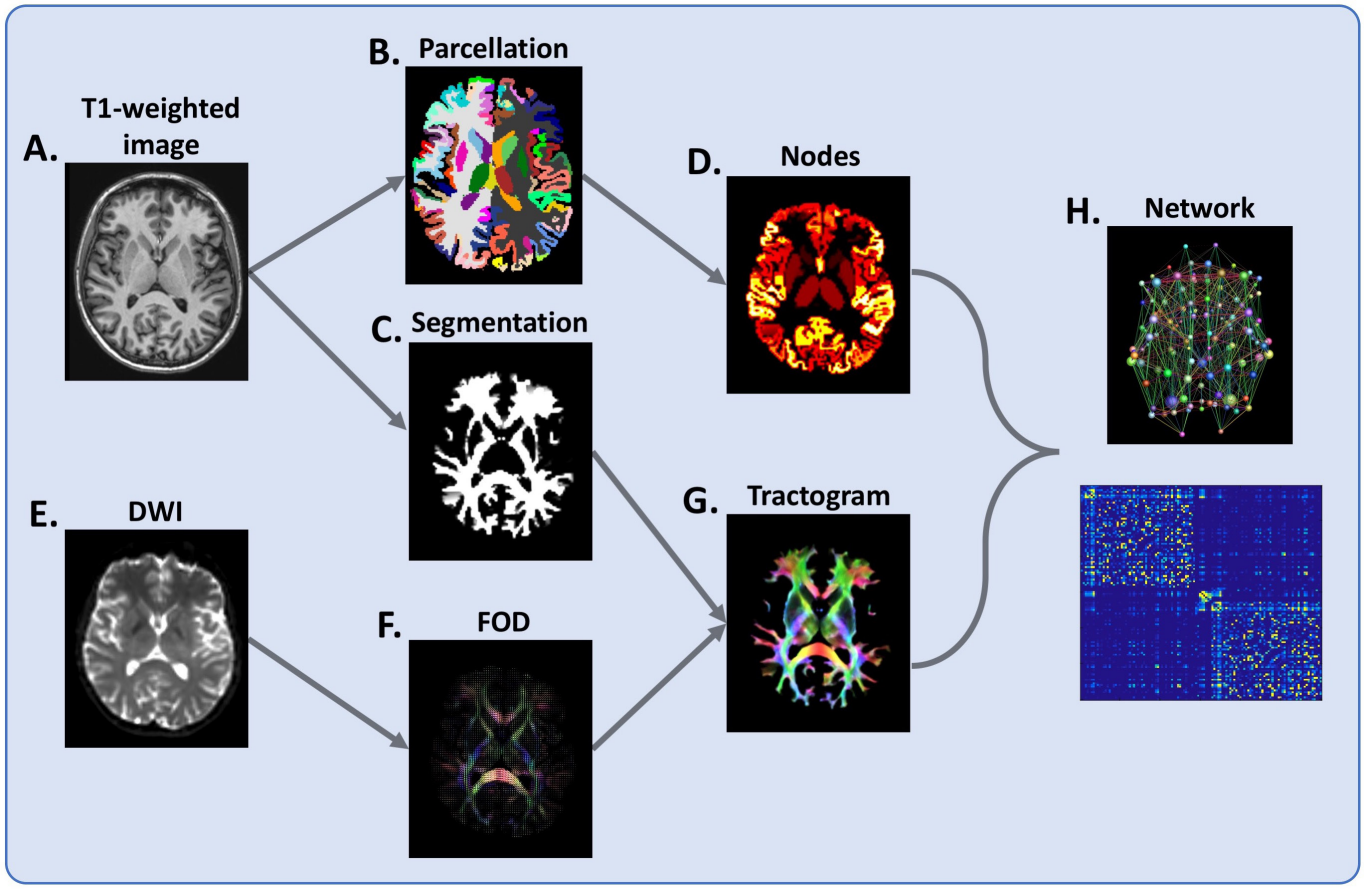
Flowchart of brain network reconstruction. For each subject, (A) T1-weighted image is segmented into grey matter (B) and white matter (C). The grey matter segmentation is parcellated into cortical and deep grey matter regions (B), which serve as network nodes (D) in the subsequent network-based analysis. From a diffusion-weighted image (DWI) (E), voxel-wise fibre orientation distribution (FOD) (F) is estimated and whole-brain tractography undertaken (G), with the white matter segmentation (C) used to prevent this from spilling into grey matter (see main text for details). Finally, nodes and tractogram are modelled into a network (H). Connections are weighted by the sum of the pairwise streamline weights.

We constructed a symmetric matrix consisting of 120 nodes. Each network edge was defined as the sum of weights of streamlines connecting a pair of nodes.^13^ Figure 1 summarises the pipeline. We extracted a range of standard network measures using TractoR^21^: Edge density, also known as connectivity, is the ratio of the connections exist relative to the number of potential connections. Global efficiency is a network integration metric that describes the information flow over the entire network while local efficiency is considered a local homolog quantifying information transfer within local networks. Finally, clustering coefficient reflects the number of connections between neighbouring nodes and is related to network segregation 22 (for further details on MRI parameters and analysis see online supplementary methods).

10.1136/jnnp-2018-318440.supp3 Supplementary data

### Statistical analysis

Statistical analysis was performed using R software (https://www.r-project.org/ V.3.3.0). For all the models, we explored whether there was a violation of normality assumption of the residuals. Data are reported as mean±SD, unless otherwise stated. P values <0.05 were considered statistically significant.

### Preliminary analysis

**Figure 2:**
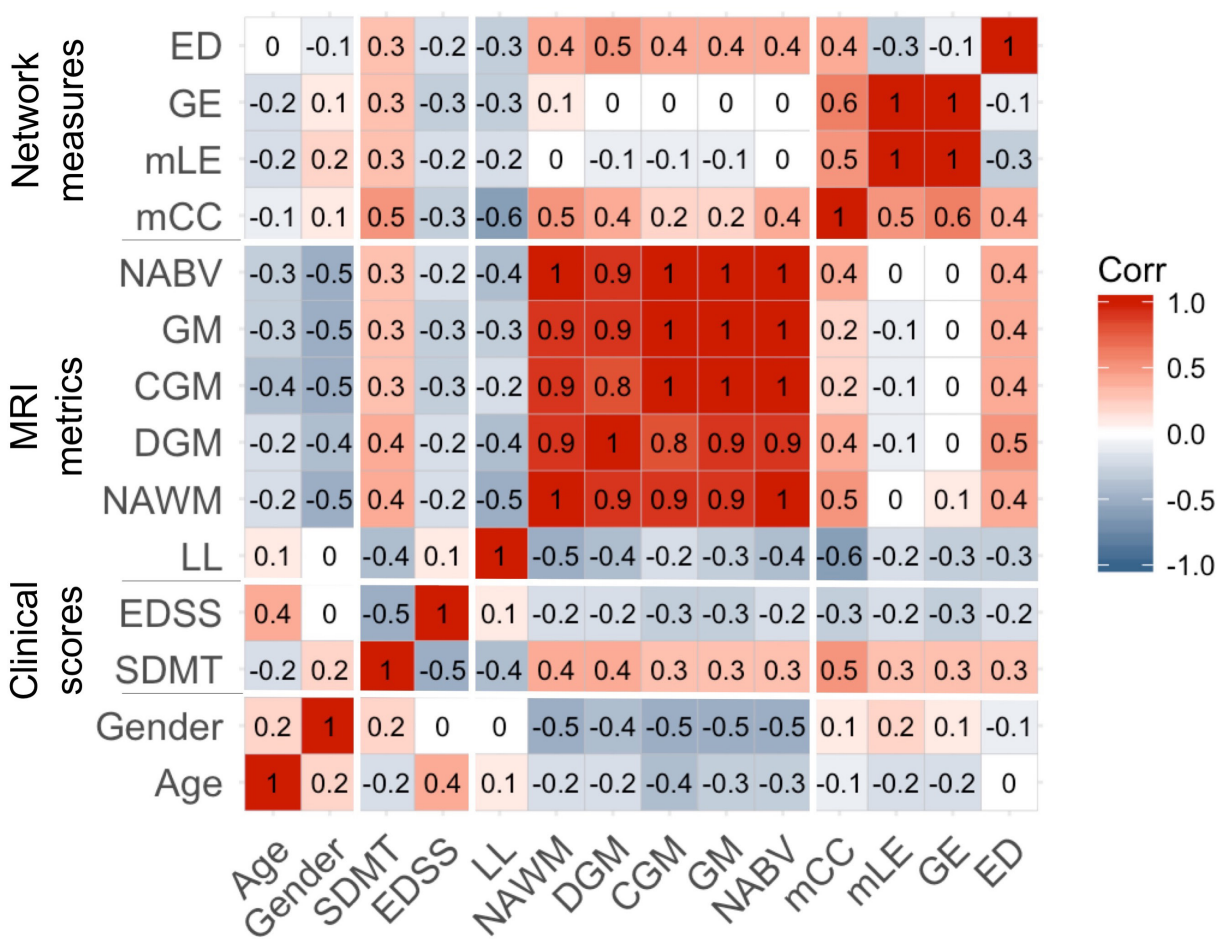
Descriptive pairwise univariable associations in patients. The reported value in each entry of the matrix corresponds to the pairwise Pearson correlation coefficient (r). Gender is a binary variable in which 0 is male and 1 female. CGM, cortical grey matter; DGM, deep grey matter; ED, Edge density; EDSS, Expanded Disability Status Scale; GE, global efficiency; GM, grey matter; LL, lesion load; mCC, mean clustering coefficient; mLE, mean local efficiency; MRI, magnetic resonance imaging; NABV, normal appearing brain volume; NAWM, normal appearing white matter; SDMT, Symbol Digit Modalities Test.

To assess network differences between subjects’ groups, ANOVA analysis was used, adjusting for age, gender, LL and total intracranial volume (TIV) to correct for head size. To explore possible associations of all the variables in patients, we used bivariate Pearson’s correlations. The variables include network metrics (edge density, global efficiency, mean local efficiency and mean clustering coefficient), atrophy measures (NABV, GM, CGM, DGM), WM damage metrics (LL), clinical scores (EDSS and SDMT) and patient age and gender (figure 2). In this study, atrophy measures and WM lesions are also referred to as MRI metrics. Volumetric differences between HC and patients with MS were also assessed (online supplementary etable 3).

### Network measures and volumetric parameters in explaining EDSS and SDMT

We performed stepwise linear regression analyses using each of the volume metrics (in turn) as independent variables and age, gender and LL as covariates to explain clinical scores (dependent variable). We also controlled for the presence of disease-modifying treatments (DMTs). We selected the best model as assessed with the adjusted R^2^ (Adj.R^2^) and then added each network metric, in turn, as an independent variable. For SDMT, we performed a post hoc analysis controlling for education level as a categorical and afterwards as a continuous variable to investigate a possible linear relationship between education level and SDMT. To assess whether the effect for each network metric in explaining disability is group dependent, we stratified the MS population based on their clinical profile by creating an interaction term, for example the product between the network metric and a categorical variable for MS subgroup (RRMS, PPMS, SPMS), ‘network metrics×MS subgroup’, that was then included in the model as an explanatory variable. For SDMT, we explored possible associations between network metrics and MRI variables in HC.

### Final models to explain EDSS and SDMT

To find the best model that explains disability, a stepwise forward selection linear regression strategy was employed. All variables of interest were sequentially added to the model and kept only if significant, culminating in two final models, one per each clinical score.

## Results

Demographic, clinical, MRI and network data from patients with MS and HC are summarised in table 1.

**Table 1:** Demographic, clinical, MRI, and network metrics

	HC (n=51)	Patients with MS (n=122)	RRMS (n=58)	PPMS (n=28)	SPMS (n=36)
Demographics					
Age, years	41±13	48±11	42±10	52±9	53±7
Gender (M/F)	25/26	36/86	18/40	10/18	8/28
Disease duration, years	–	15±10	11±8	14±7	22±10
% (n) patients of DMTs	–	58 (67)	84 (48)	13 (3)	47 (16)
% (n) patients who relapsed in the previous 2 years	–	51 (38)	68 (32)	0 (0)	24 (6)
Clinical scores					
EDSS, median	–	5.5 (0–8.5)	2 (0–7)	6 (3–8)	6.5 (4–8.5)
SDMT	65.08±8.31	45.50±13.27	51.04±14.28	42.86±9.46	39.00±10.88
MRI metrics					
NABV (cm^3^ *)*	1158±102	1042±120	1070±123	1060±122	984±93
GM (cm^3^)	679±57	625±65	641±64	632±67	593±52
CGM (cm^3^)	640±54	591±61	606±61	597±65	561±50
DGM (cm^3^)	39.00±3.39	34.18±4.02	34.86±4.09	35.41±3.50	32.12±3.54
NAWM (cm^3^)	480±49	418±59	429±62	429±60	391±45
LL (mL)	–	14.37±15.92	12.78±15.72	16.56±19.83	15.23±12.73
Network metrics					
Edge density, (%)	92.6±2.7	90.6±3.2	90.8±3.3	90.5±3.0	90.3±3.0
Global efficiency	3881±121	3783±175	3827±137	3763±196	3729±199
Mean local efficiency	3975±139	3889±200	3934±160	3868±220	3831±229
Mean clustering coefficient	247±9.2	223±18.3	227±17.2	224±20.5	217±16.8

CGM, cortical grey matter; DGM, deep grey matter; DMT, disease-modifying treatment; EDSS, Expanded Disability Status Scale; GM, grey matter; HC, healthy controls; LL, lesion load; MS, multiple sclerosis; NABV, normal-appearing brain volume; NAWM, normal-appearing white matter; PPMS, primary progressive MS; RRMS, relapsing remitting MS; SDMT, Symbol Digit Modalities Test; SPMS, secondary progressive MS.

### Differences in network metrics in MS population and subtypes

There was a significant decrease in global efficiency (regression coefficient (RC)=−71.23, p=0.016), mean local efficiency (RC=−72.53, p=0.031) and mean clustering coefficient (RC=−14.84, p<0.0001) in the whole MS group when compared with HC. For the subtypes, there was reduced global efficiency in PPMS (RC=−85.82, p=0.027) and in SPMS (RC=−145.34, p=0.0002) relative to HC and also decrease in this metric in SPMS relative to RRMS (RC=−111.90, p=0.0008). Mean local efficiency was reduced in SPMS compared with HC (RC=−158.42, p=0.0002) and to RRMS (RC=−128.21, p=0.0007). Relative to HC, mean clustering coefficient was reduced in RRMS (RC=−14.84, p<0.0001), PPMS (RC=−13.42, p<0.0001) and SPMS (RC=−20.30, p<0.0001) while relative to RRMS it was reduced in SPMS (RC=−8.30, p=0.0033). There was also a significant decrease in SPMS compared with PPMS (RC=−6.88, p=0.037). All models were adjusted for age, gender, LL and TIV (table 2).

**Table 2:** Exploratory network differences between different groups

	HC			RRMS			PPMS		
	RC	95% CI	P values	RC	95% CI	P values	RC	95% CI	P values
Edge density									
MS	−0.65	(−1.69 to 0.38)	0.210						
RRMS	−0.71	(−1.84 to 0.42)	0.219						
PPMS	−0.72	(−2.1 to 0.67)	0.310	−0.011	(−1.27 to 1.25)	0.987			
SPMS	−0.45	(−1.78 to 0.88)	0.507	0.258	(−0.93 to 1.43)	0.670	0.27	(−1.12 to 1.66)	0.707
Global efficiency									
MS	−71.23	(−129.47 to −13.00)	**0.016**						
RRMS	−33.44	(−95.11 to 28.25)	0.287						
PPMS	−85.82	(−161.6 to −9.96)	**0.027**	−52.38	(−121.08 to 16.34)	0.135			
SPMS	−145.34	(−218.38 to −72.28)	**0.0001**	−111.90	(−176.47 to −47.31)	**0.0008**	−59.52	(−135.72 to 16.67)	0.126
Mean local efficiency									
MS	−72.53	(−138.60 to −6.46)	**0.031**						
RRMS	−30.21	(−100.16 to 39.74)	0.396						
PPMS	−85.68	(−171.68 to 0.34)	0.051	−55.46	(−133.39 to 22.47)	0.162			
SPMS	−158.42	(− 241.26 to −75.58)	**0.0002**	−128.21	(−201.44 to −54.96)	**0.0007**	−72.74	(−159.16 to 13.68)	0.099
Mean clustering coefficient									
MS	−14.84	(−19.89 to −9.79)	**<** **0.0001**						
RRMS	−12.00	(−17.51 to −6.48)	**<** **0.0001**						
PPMS	−13.42	(−20.19 to −6.64)	**0.0001**	−1.42	(−7.55 to 4.73)	0.650			
SPMS	−20.30	(−26.84 to −13.76)	**<** **0.0001**	−8.30	(−14.07 to −2.52)	**0.0033**	−6.88	(−13.69 to −0.06)	**0.037**

Analysis of variance was performed. P values in bold denote statistical significance at p<0.05 when the groups on the left were compared with the reference group (top row) and adjusted for age, gender, lesion load and total intracranial volume.

HC, healthy controls; MS, multiple sclerosis; PPMS, primary progressive MS; RC, regression coefficient; RRMS, relapsing–remitting MS; SPMS, secondary progressive MS.

### Descriptive associations among study variables in patients

Pairwise associations among clinical, volume and network metrics, LL age and gender study variables are shown in figure 2. Higher LL was associated with lower connectivity (r=−0.3), lower values of global (r=−0.3) or local (r=−0.2) efficiency and reduced clustering (r=−0.6). Also, lower connectivity and lower clustering coefficient were associated with reduced volumes of NABV, GM, CGM, DGM and normal-appearing WM (NAWM) (r=between 0.2 and 0.5). Moreover, we found associations between clinical scores and network measures; for example, higher EDSS and lower SDMT scores were associated with lower connectivity values, global and local efficiency and clustering coefficient (r=between 0.2 and 05). Additionally, correlation analyses between clinical scores and volume metrics demonstrated that higher EDSS scores and lower SDMT were associated with reduced volumes of NABV, GM, CGM, DGM and NAWM (r=between 0.2 and 0.5). Higher LL was also associated with decreased SDMT (r=−0.4) but showed very little correlation with EDSS score (r=0.1). We also found that higher EDSS score is associated with lower SDMT score (r=-0.5). Gender showed weak associations with network metrics (r=between −0.1 and 0.2). For age, we found that older participants show lower values of network metrics (r=−0.1 and 0.2) except edge density that shows weak linear relationship (r<0.05).

### Statistical modelling of EDSS score

We found that NABV was the only significant independent variable of EDSS after adjusting for age, gender and LL. For each millilitre decrease in NABV, there was an increase in the EDSS score of 4.06×10^–3^ (95% CI −7.68×10^–3^ to −4.3×10^–3^, p=0.029, Adj.R^2^=0.185; table 3). We did not find any significant adjusted associations between the other volume metrics and EDSS.

**Table 3:** Stepwise linear regression of EDSS in multiple sclerosis

	Model summary+predictors	Regression coefficient	95% CI	P values
MRI metrics				
EDSS score	Adj.R^2^=0.185			
	NABV, cm^3^	−0.0041	(−0.0077 to −0.00043)	**0.029**
	Age, years	0.081	(0.044 to 0.12)	**<** **0.001**
	Female	−0.73	(−1.66 to 0.20)	0.125
MRI metrics+network measures				
EDSS score	Adj.R^2^=0.205			
	NABV, cm^3^	−0.0021	(−0.0061 to 0.0019)	0.297
	Edge density, %	−0.13	(−0.26 to −0.0014)	**0.047**
	Age, years	0.087	(0.051 to 0.12)	**<** **0.001**
	Female	−0.60	(−1.53 to 0.33)	0.202
	Adj.R^2^=0.221			
	NABV, cm^3^	−0.0037	(−0.0073 to −0.00016)	**0.041**
	Global efficiency	−0.0026	(−0.0048 to −0.00058)	**0.013**
	Age, years	0.072	(0.036 to 0.11)	**<** **0.001**
	Female	−0.52	(−1.44 to 0.40)	0.266
	Adj.R^2^=0.206			
	NABV, cm^3^	−0.0041	(−0.076 to −0.00049)	**0.026**
	mLE	−0.0019	(−0.0038 to −0.000044)	**0.045**
	Age, years	0.073	(0.036 to 0.11)	**<** **0.001**
	Female	−0.57	(−1.50 to 0.37)	0.231
	Adj.R^2^=0.229			
	NABV, cm^3^	−0.0016	(−0.005 to 0.007)	0.551
	mCC	−0.029	(−0.051 to −0.0075)	**0.008**
	Age, years	0.078	(−0.0042 to 0.0022)	**<** **0.001**
	Female	−0.30	(−1.26 to 0.66)	0.534
Final model				
EDSS score	Adj.R^2^=0.259			
	Edge density, %	−0.17	(−0.28 to −0.060)	**0.003**
	Global efficiency	−0.0031	(−0.0051 to −0.0011)	**0.003**
	Age, years	0.081	(0.047 to 0.12)	**<** **0.001**

P values in bold denote statistical significance at p<0.05.

EDSS, Expanded Disability Status Scale; NABV, normal-appearing brain volume; mCC, mean clustering coefficient; mLE, mean local efficiency.

When network metrics were added to the model reported above as independent variable, in turn, we found that they were each associated with EDSS independently of NABV. Specifically, for each percentage point decrease in edge density, there was an increase in the EDSS score of 0.13 (95% CI −0.27 to −1.49×10^–3^, p=0.047, Adj.R^2^=0.205), and for each unit decrease in global efficiency, there was an increase in EDSS of 2.67×10^–3^ (95% CI −4.75×10^–3^ to −5.81×10^–4^, p=0.013, Adj.R^2^=0.221). For each unit decrease in mean local efficiency, there was an increase in EDSS of 1.90×10^–3^ (95% CI −3.76×10^–3^ to −4.40×10^–5^, p=0.045, Adj.R^2^=0.206), and for each unit decrease in mean clustering coefficient, there was an increase in EDSS of 3.98×10^–2^ (95% CI −6.34×10^–2^ to −1.61×10^–2^, p=0.011, Adj.R^2^=0.235) (table 3). We did not find any significant difference in the effect of any of the network measures in any of the MS subgroups examined. All the above models were adjusted for age, gender, LL and DMTs.

The best model to explain EDSS using the stepwise forward selection linear regression analysis showed that lower edge density, lower global efficiency and increased participants’ age explained 26% of the variance in EDSS (table 3). The explained variance is higher compared with 18.5% for NABV alone or with 20% for global efficiency (−0.02, 95% CI −0.0049 to −0.00063, p=0.012) or with 20% for edge density (−0.16, 95% CI −0.28 to −0.035, p=0.012).

### Statistical modelling of SDMT score

We repeated the multiple linear regression analyses to explain SDMT. When only volume metrics were included, the best model fit was achieved by DGM as independent variable, showing that for each 1 cm^3^ decrease in the volume of DGM, there was a decrease in the SDMT of 1.61 (95% CI 0.79 to 2.43, p<0.001, Adj.R^2^=0.361; table 4), that is, smaller DGM volumes were associated with worse information processing speed in the whole MS group.

**Table 4:** Stepwise linear regression of SDMT in multiple sclerosis

	Model summary+predictors	Regression coefficient	95% CI	P values
MRI metrics				
SDMT score	Adj.R^2^=0.361			
	DGM, cm^3^	1.61	(0.79 to 2.43)	**<** **0.001**
	Lesion load, mL	−0.17	(−0.34 to −0.0014)	**0.048**
	Female	12.16	(5.51 to 18.82)	**<** **0.001**
MRI metrics+network measures				
SDMT score	Adj.R^2^=0.352			
	DGM, cm^3^	1.52	(0.61 to 2.43)	**0.001**
	Lesion load, mL	−0.17	(−0.34 to 0.0069)	0.059
	Edge density, (%)	0.24	(−0.75 to 1.23)	0.624
	Female	11.94	(5.18 to 18.70)	**<** **0.001**
	Adj.R^2^=0.396			
	DGM, cm^3^	1.93	(1.21 to 2.65)	**<** **0.001**
	Global efficiency	0.021	(0.0055 to 0.035)	**0.008**
	Female	10.97	(4.37 to 17.56)	**0.002**
	Adj.R^2^=0.380			
	DGM, cm^3^	2.01	(1.28 to 2.75)	**<** **0.001**
	mLE	0.015	(0.0028 to 0.028)	**0.018**
	Female	11.43	(4.79 to 18.06)	**0.001**
	Adj.R^2^=0.387			
	DGM, cm^3^	1.45	(0.63 to 2.28)	**<** **0.001**
	mCC	0.21	(0.047 to 0.38)	**0.013**
	Female	9.92	(2.98 to 16.85)	**0.006**
Final model				
SDMT score	Adj.R^2^=0.396			
	DGM, cm^3^	1.93	(1.21 to 2.65)	**<** **0.001**
	Global efficiency	0.021	(0.0055 to 0.035)	**0.008**
	Female	10.97	(4.36 to 17.56)	**0.002**

P values in bold denote statistical significance at p<0.05.

DGM, deep grey matter; SDMT, Symbol Digit Modalities Test; mCC, mean clustering coefficient; mLE, mean local efficiency.

When we added network metrics, in turn, in our multiple regression analysis that included DGM, we found that global efficiency, mean local efficiency and mean clustering coefficient were able to significantly explain additional variance in SDMT. For each unit increase in global efficiency, there was an increase in the SDMT of 0.02 (95% CI 0.01 to 0.04, p=0.008, Adj.R^2^=0.396). For each unit increase in mean local efficiency, there was an increase in the SDMT of 0.02 (95% CI 0.002 to 0.03, p=0.018, Adj.R^2^=0.380), and finally for each unit increase in mean clustering coefficient, there was an increase in the SDMT of 0.21 (95% CI 0.05 to 0.38, p=0.013, Adj.R^2^=0.387). There was no evidence of change of SDMT per percentage increase in edge density (0.44, 95% CI −0.56 to 1.44, p=0.38, Adj.R^2^=0.374; table 1). Additionally, there was no significant difference in the effect of any of the network metrics in any of the subgroups examined while the statistical models do not explain SDMT in HC. All the above models were adjusted for age, gender, LL and DMTs.

The best model to explain SDMT using the stepwise forward selection linear regression analysis showed that greater DGM volume, greater global efficiency and female gender were all associated with better information processing speed (table 1). This model explained 39.6% of the variance in SDMT scores compared with 36% for the DGM alone (1.61, 95% CI 0.79 to 2.43, p<0.001).

## Discussion

This study showed structural network topological changes within the various MS groups. We also demonstrated that markers of structural network disruption explain EDSS and SDMT scores above metrics of tissue atrophy and WM lesions.

### Structural network differences between subjects’ groups

We detected network topological changes in MS. Relative to HC, SPMS had reduced global and local efficiency, PPMS reduced global efficiency while there was no efficiency change in RRMS. These changes reflect network alterations due to diffuse WM pathology including impaired long-distance and short-distance connections, characteristics that are more prominent in the progressive types. Previous studies focused mainly on RRMS reporting decreases in this metric in structural^8 21 22^ and functional^22–24^ networks while others, in accordance with this work, found no differences.^25^ Intriguingly, increased efficiency in RRMS in the first year from onset in the absence of clinical impairment is suggestive of structural adaptations to maintain normal function.^9^ Our RRMS cohort has a relatively long disease duration with high EDSS due to accrual of baseline disability as a result of incomplete recovery from relapses explaining partly the absence of this effect. Yet, only one study considered SPMS and PPMS group reporting reduced global efficiency, in accordance with our findings.^21^ Moreover, we demonstrate reduced global and local efficiency in SPMS relative to RRMS, a result likely to reflect the neurodegenerative component in this progressive subtype.

Clustering coefficient is a ‘small-world’ metric and reduction suggests a more random architecture^26^ related to increased disability as shown in our study and elsewhere.^27^ Previous structural studies reported increased clustering coefficient in RRMS compared with HC^9 28 29^ and is thought to reflect transient compensatory changes. No change was reported in functional networks.^23 30^ Here, we report a decrease in clustering coefficient in RRMS compared with HC, in agreement with a study that investigated both structural and functional networks.^22^ We also extend these findings demonstrating reduction of this metric in the progressive phases. Clustering coefficient was further reduced in SPMS relative to RRMS and PPMS indicating that impaired local information flow is linked to the disease severity. Nonetheless, further investigations with bigger sample sizes and longitudinal study design should confirm the study findings.

### Network measures explain additional variance of disability

Whole brain atrophy is a relatively strong predictor of EDSS. Our study shows that the addition of network metrics into the model, singly and together, explains more EDSS variance, leading to our final model (table 3), according to which edge density and global efficiency explain 26% of the variance, that is 7% more compared with NABV alone (19%). Loss of connectivity could reflect neurodegeneration due to continuous inflammation^31^ while reduced global efficiency could indicate impaired structural long-range connections probably due to inflammatory activity and neuroaxonal loss.^32^ The fact that these measures integrate information beyond local tissue damage and atrophy measures may account for the increased explained variance.

SDMT was most strongly associated with DGM atrophy and WM damage.^33^ Previous structural and functional studies demonstrated the relationship between network disruption and cognitive impairment.^4 33 34^ Our study findings showed that global efficiency is associated with SDMT as previously shown and it also explained additional variance (table 1) highlighting that intact network integration is important for efficient information processing beyond participant’s education level and treatment. These findings are also consistent across WM diseases^35^ signifying the relevance of network efficiency as potential marker of cognitive disability.

MS is a heterogeneous disease. This study included patients with MS with the main disease phenotypes in order to provide a representative snapshot of structural networks throughout the entire disease course. Our regression analyses show that the behaviour of the network metrics was similar in all MS subtypes suggesting that these measures could be useful across the whole MS disease spectrum. The same statistical models did not explain SDMT in HC. This negative result is not surprising given the narrow distribution of the SDMT variable in HC compared with patients (see table 2 for mean and SD). Furthermore, due the small number of HC for which we have SDMT (n=12), these results should be interpreted with caution. Future studies could assess whether the findings presented here are replicated in other cohorts.

### Descriptive associations among study variables in patients

Our univariate associations in patients revealed some interesting patterns. Low values of network metrics were associated with clinical impairment and worse information processing speed in accordance with previous studies.^8 22 25^ Interestingly, reduced clustering showed the strongest association out of network metrics with worse SDMT indicating that network randomisation impairs information processing speed as shown previously.^27^ Our multivariate analysis though demonstrated that reduced network integration and tissue atrophy can more strongly affect SDMT performance. In line with previous work,^8^ WM lesions impair the communication between brain regions at the global and local level as demonstrated by the reduced network efficiencies. As shown in the exact same cohort, we did not find any association between WM lesions and EDSS^10^ and only weak association between WM lesions and SDMT, and this highlights the need to explore non-conventional MRI metrics to explain disability. Also, there was no association between edge density and any of the network efficiencies. Although this could be the result of wiring cost and efficiency,^26^ we argue that direct comparison between binary and weighted network is not valid.

In our approach, we used CSD to model intravoxel crossing fibres,^20^ and ACT and SIFT2 to improve connection and streamline density,^12 13^ respectively, with the assumption that the FOD amplitude corresponds to the underlying fibre density.^36^ These advanced methods improve tractogram reconstruction without the need of various scaling techniques.^14^ We also provided anatomical prior of the WM ensuring that no streamlines are incorrectly terminated in the WM due to lesions (online supplementary efigure 1). In fact, we identified an association between LL and connectivity, but this correlation is not that high (r=−0.3), which highlights that our current approach is not overly influenced by lesions.

10.1136/jnnp-2018-318440.supp1 Supplementary data

### Limitations and future directions

This study has several limitations. In our approach, we applied techniques to address some of the reconstruction biases and to ensure that no streamlines were abnormally terminated in WM.^12–14^ However, histological validation studies are required to make direct links between imaging measures and underlying pathology. Additionally, the cross-sectional design of the study does not allow to determine the clinical relevance of network measures over time. Moreover, we used SDMT scores for approximately half of MS cohort (n=60 vs n=122), but this subcohort had similar proportions of MS subgroups to the whole cohort (online supplementary etable 1). Also, the effects of fatigue, depression and anxiety can be investigated in future studies with larger cohorts. A post hoc analysis revealed depression and anxiety scores showed mild correlations with SDMT whereas fatigue did not. It is difficult to investigate their influences in our cohort as the HADS and fatigue scores were not collected in all subjects with SDMT. Finally, although the effect of cortical lesions in clinical scores is limited,^37^ it is possible that they may influence our study outcomes.

The study findings could provide the basis for future work. There are different scales that we could study MS from, including micro, meso and macro scales.^38^ Network analysis offers a framework at the macroscale to study whole brain connectivity patterns beyond focal pathology while TBSS, for example, is currently considered a leading technique for the voxel-wise DTI analysis.^4^ Future investigations could focus in the comparison between scales and their link with clinical outcome. Additionally, further studies could follow a subnetwork or nodal rather than global network analysis and perhaps derive integrative measures of structural and functional networks and investigate if these parameters explain additional variance.

## Conclusion

In conclusion, we found distinct network organisation in the various groups. Also, network metrics and in particular global efficiency explains disability over and above non-network metrics supporting the relevance of intact long-distance connexions mainly, to maintain normal function. These results highlight the potential of network parameters as biomarkers for disease diagnosis, prognosis and in clinical trials.

## References

[1] Mahad DH, Trapp BD, Lassmann H Pathological mechanisms in progressive multiple sclerosis. Lancet Neurol 2015;14:183–93. 10.1016/S1474-4422(14)70256-X 25772897

[2] Fisniku LK, Brex PA, Altmann DR, et al Disability and T2 MRI lesions: a 20-year follow-up of patients with relapse onset of multiple sclerosis. Brain 2008;131(Pt 3):808–17. 10.1093/brain/awm329 18234696

[3] Muhlert N, Sethi V, Cipolotti L, et al The grey matter correlates of impaired decision-making in multiple sclerosis. J Neurol Neurosurg Psychiatry 2015;86:530–6. 10.1136/jnnp-2014-308169 25006208 PMC4413680

[4] Dineen RA, Vilisaar J, Hlinka J, et al Disconnection as a mechanism for cognitive dysfunction in multiple sclerosis. Brain 2009;132(Pt 1):239–49. 10.1093/brain/awn275 18953055

[5] Rocca MA, Amato MP, De Stefano N, et al Clinical and imaging assessment of cognitive dysfunction in multiple sclerosis. Lancet Neurol 2015;14:302–17. 10.1016/S1474-4422(14)70250-9 25662900

[6] Kurtzke JF Rating neurologic impairment in multiple sclerosis: an expanded disability status scale (EDSS). Neurology 1983;33:1444–52. 10.1212/WNL.33.11.1444 6685237

[7] Fornito A, Zalesky A, Breakspear M The connectomics of brain disorders. Nat Rev Neurosci 2015;16:159–72. 10.1038/nrn3901 25697159

[8] Shu N, Liu Y, Li K, et al Diffusion tensor tractography reveals disrupted topological efficiency in white matter structural networks in multiple sclerosis. Cereb Cortex 2011;21:2565–77. 10.1093/cercor/bhr039 21467209

[9] Fleischer V, Gröger A, Koirala N, et al Increased structural white and grey matter network connectivity compensates for functional decline in early multiple sclerosis. Mult Scler 2017;23:432–41. 10.1177/1352458516651503 27246143

[10] Pardini M, Yaldizli Ö, Sethi V, et al Motor network efficiency and disability in multiple sclerosis. Neurology 2015;85:1115–22. 10.1212/WNL.0000000000001970 26320199 PMC4603887

[11] Sotiropoulos SN, Zalesky A Building connectomes using diffusion MRI: why, how and but. NMR Biomed 2017:e3752 10.1002/nbm.3752 28654718 PMC6491971

[12] Smith RE, Tournier JD, Calamante F, et al Anatomically-constrained tractography: improved diffusion MRI streamlines tractography through effective use of anatomical information. Neuroimage 2012;62:1924–38. 10.1016/j.neuroimage.2012.06.005 22705374

[13] Smith RE, Tournier JD, Calamante F, et al SIFT2: enabling dense quantitative assessment of brain white matter connectivity using streamlines tractography. Neuroimage 2015;119:338–51. 10.1016/j.neuroimage.2015.06.092 26163802

[14] Yeh CH, Smith RE, Liang X, et al Correction for diffusion MRI fibre tracking biases: the consequences for structural connectomic metrics. Neuroimage 2016;142:150–62. 10.1016/j.neuroimage.2016.05.047 27211472

[15] Lublin FD, Reingold SC Defining the clinical course of multiple sclerosis: results of an international survey. National Multiple Sclerosis Society (USA) advisory committee on clinical trials of new agents in multiple sclerosis. Neurology 1996;46:907–11. 8780061 10.1212/wnl.46.4.907

[16] Bhushan C Correcting susceptibility-induced distortion in diffusion-weighted MRI using constrained nonrigid registration. Signal Inf Process Assoc Annu Summit Conf APSIPA Asia Pac, 2012. PMC4708288 26767197

[17] Prados F, Cardoso MJ, Kanber B, et al A multi-time-point modality-agnostic patch-based method for lesion filling in multiple sclerosis. Neuroimage 2016;139:376–84. 10.1016/j.neuroimage.2016.06.053 27377222 PMC4988790

[18] Cardoso MJ, Modat M, Wolz R, et al Geodesic information flows: spatially-variant graphs and their application to segmentation and fusion. IEEE Trans Med Imaging 2015;34:1976–88. 10.1109/TMI.2015.2418298 25879909

[19] Andersson JLR, Sotiropoulos SN An integrated approach to correction for off-resonance effects and subject movement in diffusion MR imaging. Neuroimage 2016;125:1063–78. 10.1016/j.neuroimage.2015.10.019 26481672 PMC4692656

[20] Tournier JD, Calamante F, Connelly A Robust determination of the fibre orientation distribution in diffusion MRI: non-negativity constrained super-resolved spherical deconvolution. Neuroimage 2007;35:1459–72. 10.1016/j.neuroimage.2007.02.016 17379540

[21] Kocevar G, Stamile C, Hannoun S, et al Graph theory-based brain connectivity for automatic classification of multiple sclerosis clinical courses. Front Neurosci 2016;10:478 10.3389/fnins.2016.00478 27826224 PMC5078266

[22] Shu N, Duan Y, Xia M, et al Disrupted topological organization of structural and functional brain connectomes in clinically isolated syndrome and multiple sclerosis. Sci Rep 2016;6:29383 10.1038/srep29383 27403924 PMC4941534

[23] Rocca MA, Valsasina P, Meani A, et al Impaired functional integration in multiple sclerosis: a graph theory study. Brain Struct Funct 2016;221:115–31. 10.1007/s00429-014-0896-4 25257603

[24] Liu Y Functional brain network alterations in clinically isolated syndrome and multiple sclerosis: a graph-based connectome study. Radiology 2017;282:534–41. 27541686 10.1148/radiol.2016152843

[25] Llufriu S, Martinez-Heras E, Solana E, et al Structural networks involved in attention and executive functions in multiple sclerosis. Neuroimage Clin 2017;13:288–96. 10.1016/j.nicl.2016.11.026 28050344 PMC5192049

[26] Bullmore E, Sporns O The economy of brain network organization. Nat Rev Neurosci 2012;13:336–49. 10.1038/nrn3214 22498897

[27] Douw L, Schoonheim MM, Landi D, et al Cognition is related to resting-state small-world network topology: an magnetoencephalographic study. Neuroscience 2011;175:169–77. 10.1016/j.neuroscience.2010.11.039 21130847

[28] Muthuraman M, Fleischer V, Kolber P, et al Structural brain network characteristics can differentiate CIS from early RRMS. Front Neurosci 2016;10:14 10.3389/fnins.2016.00014 26869873 PMC4735423

[29] Tewarie P, Steenwijk MD, Tijms BM, et al Disruption of structural and functional networks in long-standing multiple sclerosis. Hum Brain Mapp 2014;35:5946–61. 10.1002/hbm.22596 25053254 PMC6869798

[30] Schoonheim MM, Hulst HE, Landi D, et al Gender-related differences in functional connectivity in multiple sclerosis. Mult Scler 2012;18:164–73. 10.1177/1352458511422245 21908484

[31] Friese MA, Schattling B, Fugger L Mechanisms of neurodegeneration and axonal dysfunction in multiple sclerosis. Nat Rev Neurol 2014;10:225–38. 10.1038/nrneurol.2014.37 24638138

[32] Mangeat G, Badji A, Ouellette R, et al Changes in structural network are associated with cortical demyelination in early multiple sclerosis. Hum Brain Mapp 2018;39:2133–46. 10.1002/hbm.23993 29411457 PMC5895520

[33] Tewarie P, Schoonheim MM, Schouten DI, et al Functional brain networks: linking thalamic atrophy to clinical disability in multiple sclerosis, a multimodal fMRI and MEG study. Hum Brain Mapp 2015;36:603–18. 10.1002/hbm.22650 25293505 PMC6869443

[34] Schoonheim MM, Meijer KA, Geurts JJ Network collapse and cognitive impairment in multiple sclerosis. Front Neurol 2015;6: :82 10.3389/fneur.2015.00082 25926813 PMC4396388

[35] Tuladhar AM, van Uden IW, Rutten-Jacobs LC, et al Structural network efficiency predicts conversion to dementia. Neurology 2016;86:1112–9. 10.1212/WNL.0000000000002502 26888983 PMC4820137

[36] Raffelt D, Tournier JD, Rose S, et al Apparent Fibre Density: a novel measure for the analysis of diffusion-weighted magnetic resonance images. Neuroimage 2012;59:3976–94. 10.1016/j.neuroimage.2011.10.045 22036682

[37] van de Pavert SH, Muhlert N, Sethi V, et al DIR-visible grey matter lesions and atrophy in multiple sclerosis: partners in crime? J Neurol Neurosurg Psychiatry 2016;87:461–7. 10.1136/jnnp-2014-310142 25926483 PMC4853554

[38] Cercignani M, Gandini Wheeler-Kingshott C From micro- to macro-structures in multiple sclerosis: what is the added value of diffusion imaging. NMR Biomed 2018:e3888 10.1002/nbm.3888 29350435

[39] Clayden J TractoR: magnetic resonance imaging and tractography with R. JSS, 2011.

[40] Rubinov M, Sporns O Complex network measures of brain connectivity: uses and interpretations. Neuroimage 2010;52:1059–69. 10.1016/j.neuroimage.2009.10.003 19819337

