# Supplementary material for: Structural network disruption markers explain disability in multiple sclerosis
Source: J Neurol Neurosurg Psychiatry. 2018 Nov 22;90(2):219–26. doi: 10.1136/jnnp-2018-318440 (PMC6518973; doi:10.1136/jnnp-2018-318440)
Supplement: Supplementary data [file jnnp-2018-318440supp002.pdf]

Supplemental Results

eTable 1: Characteristics of the participants with and without SDMT score

	Whole MS population (n = 122)	MS Population with SDMT (n = 60)	MS Population without SDMT (n = 62)	p value
**Subtypes**	RRMS = 58<br>PPMS = 28<br>SPMS = 36	RRMS = 28<br>PPMS = 14<br>SPMS = 18	RRMS = 29<br>PPMS = 15<br>SPMS = 18	0.98[a]
**Age, years**	48 ± 11	47 ± 11	49 ± 10	0.48[b]
**Gender (M/F)**	36/86	18/42	18/44	0.99[a]
**Disease duration, years**	15 ± 10	16 ± 11	14 ± 8	0.29[b]
**EDSS, median**	5.5 (0-8.5)	4.5 (1.0-8.5)	6 (0 - 8.5)	0.07[b]

Values listed are mean ± standard deviation (SD)

Comparison was performed between the MS population with and without SDMT

[a] chi Square test

[b] Student t-test for independent samples

Abbreviations: MS = multiple sclerosis; RRMS = relapsing-remitting MS; PPMS = primary progressive MS; SPMS = secondary progressive MS; EDSS = Expanded Disability Scale Status; SDMT = Symbol Digit Modality Test;

eTable 2: Depression and fatigue levels of the participants with SDMT scores

	HC	MS patients	RRMS	PPMS	SPMS
**Anxiety**					
**Mean ± SD**	4.72 ± 3.90	6.31 ± 3.68	6.28 ± 3.72	5.54 ± 4.03	7.08 ± 3.42
**% (no) subjects**	92 (11)	78 (47)	93 (26)	73 (11)	67 (12)
**Depression**					
**Mean ± SD**	2.74 ± 2.46	5.92 ± 3.46	5.43 ± 3.56	6.25 ± 3.88	6.83 ± 2.79
**% (no) subjects**	92 (11)	78 (47)	93 (26)	73 (11)	67 (12)
**Fatigue**					
**Mean ± SD**	3.05 ± 2.75	4.36 ± 2.51	4.03 ± 2.18	4.40 ± 2.67	4.97 ± 3.06
**% (no) subjects**	92 (11)	78 (47)	93 (26)	73 (11)	67 (12)

Values listed are mean ± standard deviation (SD). Fatigue was measured using a 10cm Visual Analogue Scale. Depression and Anxiety scores were recorded using the Hospital Anxiety and Depression Scale.

Abbreviations: MS = multiple sclerosis; RRMS = relapsing-remitting MS; PPMS = primary progressive MS; SPMS = secondary progressive MS;

A. Volumetric differences between HC and MS patients and subtypes

There was a significant decrease in the volumes of BV, GM, CGM, DGM and WM (all $P<0.05$) in patients compared with HC after adjusting age, gender and LL. Examining the MS subtypes, RRMS and SPMS had lower volumes (BV, GM, WM, CGM, DGM) adjusted for age, gender and LL (all $P<0.05$) when compared with HCs. PPMS group also had lower BV, DGM and WM (all $P<0.05$) whereas the decrease in CGM volume vs HCs showed borderline significance ($P=0.063$ adjusted for age, gender and LL). See **eTable 3 (supplemental)**.

eTable 3: Between group differences in MRI metrics

	Volume metrics											
	**MS patients (n = 122)**			**RRMS (n = 58)**			**PPMS (n = 28)**			**SPMS (n = 36)**		
	**RC**	**95% CI**	**P-value**	**RC**	**95% CI**	**P-value**	**RC**	**95% CI**	**P-value**	**RC**	**95% CI**	**P-value**
**BV (cm^3)**	-79.51	(-112.40 to -46.62)	**<0.001**	-64.86	(-100.57 to -29.15)	**<0.001**	-69.33	(-115.35 to 23.31)	**0.034**	-126.86	(-170.95 to 82.77)	**<0.001**
**GM (cm^3)**	-30.67	(-47.68 to -13.65)	**<0.001**	-24.80	(-43.40 to -6.19)	**0.0093**	-24.21	(-48.19 to -0.23)	**0.048**	-51.77	(-74.74 to -28.80)	**<0.001**
**CGM (cm^3)**	-26.97	(-43.08 to 10.85)	**0.0012**	-21.36	(-39.01 to 3.72)	**0.018**	-21.59	(-44.32 to 1.15)	0.063	-46.42	(-68.20 to -24.64)	**<0.001**
**DGM (cm^3)**	-3.70	(-4.84 to -2.55)	**<0.001**	-3.43	(-4.67 to 2.20	**<0.001**	-2.62	(-4.21 to -1.03)	**0.0014**	-5.35	(-6.87 to -3.83)	**<0.001**
**WM (cm^3)**	-26.97	(-43.08 to -10.85)	**0.0012**	-27.26	(-43.72 to -10.81)	**0.0013**	-28.60	(-49.80 to -7.39)	**0.0085**	-59.88	(-80.20 to -39.56)	**<0.001**
**LL[a] (mL)**		-			-		3.78	(-3.50 to 11.06)	0.306	2.46	(-4.26 to 9.17)	0.470

Abbreviations: MS = multiple sclerosis; RRMS = relapsing-remitting MS; PPMS = primary progressive MS; SPMS = secondary progressive MS; LL = Lesion load; BV = brain volume; GM = grey matter; CGM = cortical grey matter; DGM = deep grey matter; WM = white matter; RC = regression coefficient; CI = confidence intervals

P-values in bold denote statistical significance at P < 0.05 when compared to controls and adjusted for age, gender and lesion load

[a] Lesion load was compared against relapsing-remitting group

eFigure 1: Effect of lesions on tractography

Color-coded line at the GM-WM interface overlaid on diffusion weighted image without directional weighting (B0) corresponds to the streamline termination points in **(A), (B), (C), (D)** and **(E)**. In **(B)** there are streamlines terminated in a lesion compared to **(A)** as shown by the red arrow. The red arrow points to a lesion **(C)**, to a lesion overlapped with lesion mask (orange; **D**) and to lesion overlapped with lesion mask and streamline termination points **(E).** In our study, we made sure that no streamlines are terminated in lesions. The effect of streamline termination and network measures is beyond the scope of this paper.
