# Supplementary material for: Structural network disruption markers explain disability in multiple sclerosis
Source: J Neurol Neurosurg Psychiatry. 2018 Nov 22;90(2):219–26. doi: 10.1136/jnnp-2018-318440 (PMC6518973; doi:10.1136/jnnp-2018-318440)
Supplement: Supplementary data [file jnnp-2018-318440supp003.pdf]

Supplemental Methods

A. Participants

The study included 58 RRMS (40F, mean age 49 ± 12 years), 28 PPMS (18F, mean age 46 ± 9 years) and 36 SPMS (28F, mean age 52 ± 7 years) patients who had not experienced relapses within the preceding 4 weeks. Fifty-one healthy controls (HCs; 26F, mean age [±SD] 41 ± 13 years) who had no known neurological or psychiatric disorder were also recruited. All MS patients underwent MRI scans and neurological assessment using EDSS [1] at the time of participation in the study. SDMT (Symbol Digit Modalities Test) was also used to assess information processing speed and visual attention [2] in a subset of MS participants (n=60) for whom we had SDMT scores (**eTable 1 supplemental results**). SDMT was used to screen for cognitive impairment [3]. Levels of fatigue and depression were also assessed as previously described [4] and reported in the **eTable 2 (supplemental results)**. Clinical and demographic data for the whole MS group and the RRMS, PPMS and SPMS phenotypes are summarised in **Table 1**.

B. MRI data acquisition

MRI data were acquired using a Philips Achieva 3T MR scanner (Philips Healthcare, Best, Netherlands) with a 32-channel head coil. The whole brain High Angular Resolution Diffusion Imaging (HARDI) scan consisted of a cardiac-gated spin-echo (SE) sequence with echo planar imaging (EPI) readout: TR = 4000 ms; TE = 68 ms; 72 axial slices with an isotropic resolution of 2x2x2 mm^3; 61 volumes with non-collinear diffusion gradients (b-value of 1200 s mm^{-2}) and 7 volumes without directional weighting. 3D sagittal T1-weighted scans were acquired using a fast-field echo scan: TR=6.9 ms; TE=3.1 ms; inversion time=824.5 ms, resolution = 1x1x1 mm^3. For each subject, dual-echo proton density/T2-weighted axial oblique-scans

aligned with the anterior to posterior commissure were also acquired: TR=3500 ms, TE=19/85 ms, and 50 axial slices, resolution = 1x1x3 mm^3, field of view 240x180 mm^2. All data were acquired with slices aligned with the anterior commissure (AC) – posterior commissure (PC) line to minimise the effect of head positioning on data analysis

C. Structural imaging processing

Anatomical T1-weighted images were bias field corrected using the N4 algorithm [5]. For WM lesion detection, T2-hyperintense lesions were manually delineated by two experienced raters (SvdP and DC) from the PD-T2-weighted scans using JIM (v6.0, Xinapse Systems, Aldwincle, UK).

Registration between T1-weighted and diffusion-weighted images

A non-rigid transformation was performed to register the subject's non-filled T1-weighted image to the corresponding diffusion-weighting image (DWI) using BrainSuite [6]. The target volume was the first b=0 image after DWI pre-processing, resulting in a structural image of resolution 2x2x2 mm^3. The purpose of registering the structural images to the diffusion images at this stage is two-fold: a) matching the voxel dimensions and positions of the T1-scan to that of DWI means that any subsequent image derived from the anatomical scan will be inherently aligned to the DWI; and b) aligning the anatomical image to the DWI and not the other way around ensures that a re-orientation of the gradient direction is not required.

Tissue segmentation and parcellation

We non-rigidly transformed the lesions to DWI space and then filled the T1-weighted images in this space using a modality-agnostic patch-based method [7]. The reason that we registered we registered the T1-image in DWI space before lesion filling so that we matched all the anatomical features between the two modalities incusing lesions. Hence, we ensured that the non-rigid registration was not affected by the lesion filling. The filled T1-weighted images

were then segmented into cortical grey matter, white matter, deep grey matter, brainstem and cerebrospinal fluid (CSF) and parcellated into anatomically distinct regions according to Desikan–Killiany–Tourville atlas protocol using the GIF framework [8]. This method has been previously used in different neurological diseases such as MS [9], dementia [10] and epilepsy [11] GIF is freely available as web-service at http://cmictig.cs.ucl.ac.uk/niftyweb [12]. We then estimated the volumes of the various tissue types (NABV (normal appearing brain volume (BV)), GM, CGM (cortical GM), DGM (deep GM)). Reduction of these volumes reflects atrophy. LL (lesion load) was also computed as a measure of WM focal damage.

D. Diffusion-weighted imaging processing

B0 registration, eddy current and susceptibility induced correction

The mean b0 image was rigid registered to the first b0 image. Then, the same rigid transformation was applied to the 61 DWI volumes. FSL v5.0.9 was used on the DWI data to correct for eddy current and head motion [13]. We also corrected for susceptibility induced distortions caused by EPI sequences using BrainSuite v.15b. This method uses the T1-weighted image as the registration-template to correct the diffusion data [6].

Model response function and Constrained Spherical Deconvolution

For the subsequent steps, we used MRtrix3 v0.3.14. We estimated the response function [14], the signal expected from a voxel that contains a single coherent fibre bundle, and then we performed constrained spherical deconvolution (CSD) [15, 16] to estimate the voxel-wise fibre orientation distribution (FOD).

Whole-brain streamline tractography

For each subject, 10^7 streamlines were generated. For the probabilistic tractography, the iFOD2 algorithm [17] was employed using the default parameters – step size=1.25 mm, maximum

length=250 mm, implementing the anatomically constrained tractography (ACT) framework [18]. Spherical-deconvolution informed filtering of tractograms (SIFT2) was applied to the generated tractograms to modulate the contribution of each streamline to the relevant edge [19]. In this way the streamline count is reflective of the underlying fibre density at the local level. When looking at the connection density of a particular pathway, this interpretation remains such that a larger region is likely to be intersected by a greater number of streamlines. In fact, Yeh, Smith [20] showed that the application of ACT and SIFT2 (both techniques were also applied in our study) improves the biological accuracy of the reconstructed connectome while other scaling methods provide only incomplete correction.

E. Network reconstruction

GM parcellations constituted the network nodes, 120 in total. Each network edge was defined as the sum of weights of streamlines connecting a pair of nodes [19]. The pipeline is summarised in **Fig. 1**. To assess the network topology, we extracted the following network measures:

<u>Edge Density:</u> also known as connectivity, this is defined as the percentage of connections that exist relative to the potential number of network connections [21].

<u>Global efficiency:</u> is the average of the inverse of the distance matrix of the entire network matrix [22]. It is a measure of the overall information transfer efficiency across the whole network.

<u>Local efficiency</u>: similar to global efficiency, it is defined as the average of the inverse distance matrix but in a sub-cluster of the network [22]. It is considered as a measure of the local information flow. As this is a node-specific measure we average over all the nodes to get the mean local efficiency metric.

Clustering coefficient: is also a node-specific measure which describes local organisation reflecting the number of connections between the neighbours of each node [23]. Averaging over all the nodes provides the mean clustering coefficient.

The metrics were derived using the TractoR [24] package. In this study, we used the weighted forms of the graph-derived metrics, except for density, which by definition is derived from a binary network.

Bibliography for supplemental methods

1. Kurtzke, J.F., *Rating neurologic impairment in multiple sclerosis: an expanded disability status scale (EDSS).* Neurology, 1983. **33**(11): p. 1444-52.
2. Van Schependom, J., et al., *The Symbol Digit Modalities Test as sentinel test for cognitive impairment in multiple sclerosis.* Eur J Neurol, 2014. **21**(9): p. 1219-25, e71-2.
3. Parmenter, B.A., et al., *Screening for cognitive impairment in multiple sclerosis using the Symbol digit Modalities Test.* Mult Scler, 2007. **13**(1): p. 52-7.
4. Zigmond, A.S. and R.P. Snaith, *The hospital anxiety and depression scale.* Acta Psychiatr Scand, 1983. **67**(6): p. 361-70.
5. Tustison, N.J., et al., *N4ITK: improved N3 bias correction.* IEEE Trans Med Imaging, 2010. **29**(6): p. 1310-20.
6. Bhushan, C., et al., *Correcting Susceptibility-Induced Distortion in Diffusion-Weighted MRI using Constrained Nonrigid Registration.* Signal Inf Process Assoc Annu Summit Conf APSIPA Asia Pac, 2012. **2012**.
7. Prados, F., et al., *A multi-time-point modality-agnostic patch-based method for lesion filling in multiple sclerosis.* Neuroimage, 2016. **139**: p. 376-384.
8. Cardoso, M.J., et al., *Geodesic Information Flows: Spatially-Variant Graphs and Their Application to Segmentation and Fusion.* IEEE Trans Med Imaging, 2015. **34**(9): p. 1976-88.
9. Eshaghi, A., et al., *Deep grey matter volume loss drives disability worsening in multiple sclerosis.* Ann Neurol, 2018.
10. Premi, E., et al., *Cognitive reserve and TMEM106B genotype modulate brain damage in presymptomatic frontotemporal dementia: a GENFI study.* Brain, 2017. **140**(6): p. 1784-1791.
11. Taylor, N.P., et al., *The impact of epilepsy surgery on the structural connectome and its relation to outcome.* Neurons and Cognition, 2017.
12. Prados, F., et al. *NiftyWeb: web based platform for image processing on the cloud*. in *24th Scientific Meeting and Exhibition of the International Society for Magnetic Resonance in Medicine (ISMRM)*. 2016. ISMRM.
13. Andersson, J.L. and S.N. Sotiropoulos, *An integrated approach to correction for off-resonance effects and subject movement in diffusion MR imaging.* Neuroimage, 2016. **125**: p. 1063-78.
14. Tax, C.M., et al., *Recursive calibration of the fiber response function for spherical deconvolution of diffusion MRI data.* Neuroimage, 2014. **86**: p. 67-80.
15. Tournier, J.D., F. Calamante, and A. Connelly, *Robust determination of the fibre orientation distribution in diffusion MRI: non-negativity constrained super-resolved spherical deconvolution.* Neuroimage, 2007. **35**(4): p. 1459-72.
16. Tournier, J.D., et al., *Direct estimation of the fiber orientation density function from diffusion-weighted MRI data using spherical deconvolution.* Neuroimage, 2004. **23**(3): p. 1176-85.
17. Tournier, J.D., F. Calamante, and A. Connelly, *Improved probabilistic streamlines tractography by 2nd order integration over fibre orientation distributions.* Joint Annual Meeting ISMRM-ESMRMB 2010.
18. Smith, R.E., et al., *Anatomically-constrained tractography: improved diffusion MRI streamlines tractography through effective use of anatomical information.* Neuroimage, 2012. **62**(3): p. 1924-38.

19. Smith, R.E., et al., *SIFT2: Enabling dense quantitative assessment of brain white matter connectivity using streamlines tractography.* Neuroimage, 2015. **119**: p. 338-51.
20. Yeh, C.H., et al., *Correction for diffusion MRI fibre tracking biases: The consequences for structural connectomic metrics.* Neuroimage, 2016. **142**: p. 150-162.
21. Kaiser, M., *A tutorial in connectome analysis: topological and spatial features of brain networks.* Neuroimage, 2011. **57**(3): p. 892-907.
22. Latora, V. and M. Marchiori, *Efficient behavior of small-world networks.* Phys Rev Lett, 2001. **87**(19): p. 198701.
23. Watts, D.J. and S.H. Strogatz, *Collective dynamics of 'small-world' networks.* Nature, 1998. **393**(6684): p. 440-2.
24. Clayden, J., et al., *TractoR: Magnetic Resonance Imaging and Tractography with R.* JSS, 2011.
