# Supplementary figures and images for: Structural network disruption markers explain disability in multiple sclerosis
Source: J Neurol Neurosurg Psychiatry. 2018 Nov 22;90(2):219–26. doi: 10.1136/jnnp-2018-318440 (PMC6518973; doi:10.1136/jnnp-2018-318440)

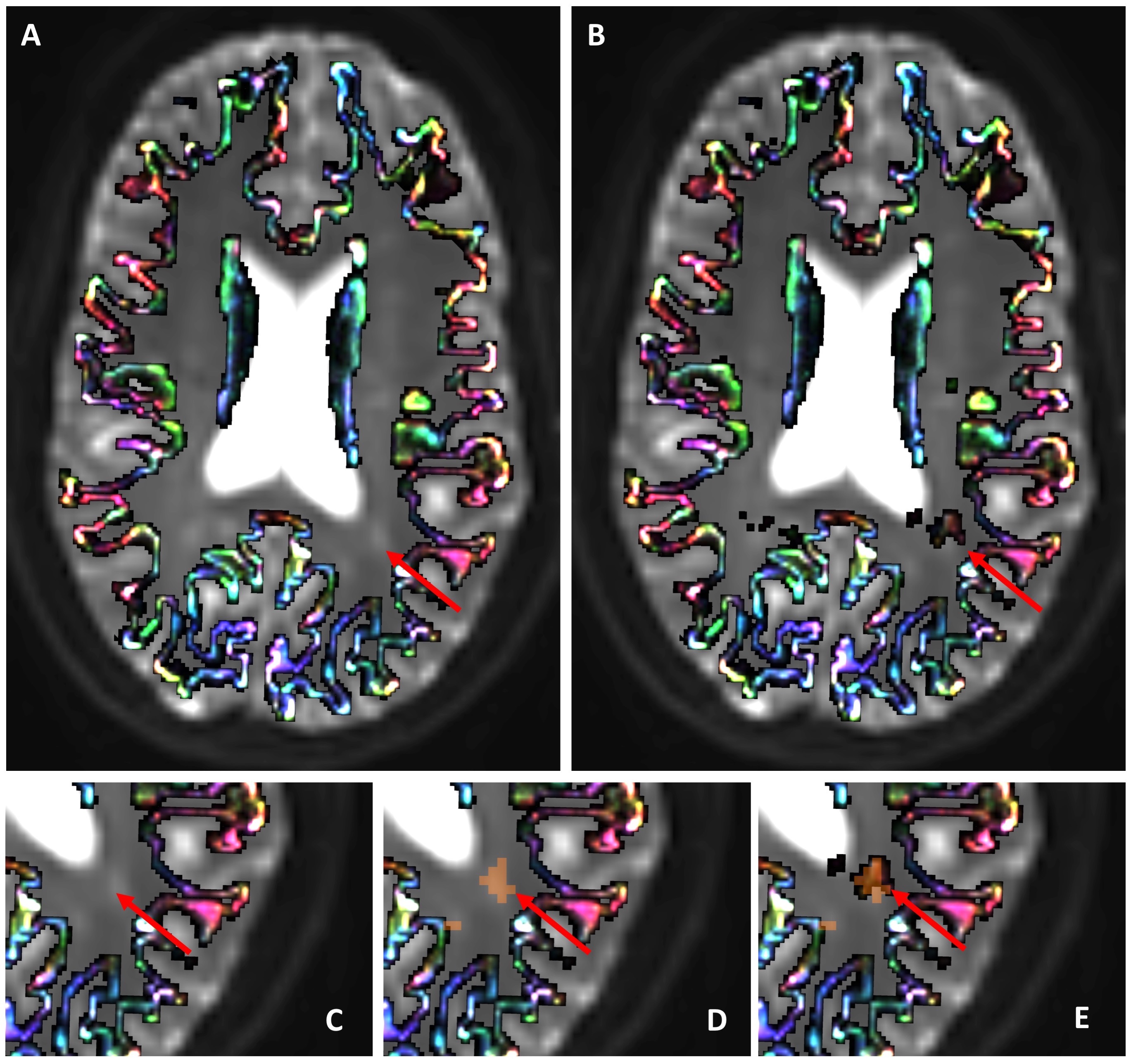

Supplement: Supplementary data [file jnnp-2018-318440supp001.jpg]
